# The Impact of REM Sleep Deprivation on ER Stress and Alzheimer-Like Pathology: Therapeutic Potential of Melatonin

**DOI:** 10.1007/s11064-026-04696-9

**Published:** 2026-02-18

**Authors:** A. Çakır, S. Şehzade, C. Koç, S. Çilingir, D. Acar, G. Süyen, A. Bican Demir, N. Kahveci

**Affiliations:** 1 Faculty of Medicine, Department of Physiology, Bursa Uludağ University, University Street, Bursa, 16059 Turkey; 2 Experimental Animal Breeding and Research Unit (DENHAB), Bursa Uludağ University, University Street, Bursa, 16059 Turkey; 3 Graduate School of Health Sciences, Department of Medicine- Physiology, Bursa Uludağ University, University Street, Bursa, 16059 Turkey; 4 Faculty of Medicine, Department of Pharmacology, Bursa Uludağ University, University Street, Bursa, 16059 Turkey; 5 Acıbadem University Technology Transfer Office, Kayışdağı Avenue, Istanbul, 34638 Turkey; 6 Graduate School of Health Sciences, Department of Physiology, Acıbadem University, Kayışdağı Avenue, Istanbul, 34638 Turkey; 7 Faculty of Medicine, Department of Physiology, Acıbadem University, Kayışdağı Avenue, Istanbul, 34638 Turkey; 8 Faculty of Medicine, Department of Neurology, Bursa Uludağ University, University Street, Bursa, 16059 Turkey

**Keywords:** Sleep deprivation, Alzheimer’s disease, Endoplasmic reticulum stress, Melatonin, 4-PBA, Unfolded protein response

## Abstract

REM sleep deprivation (REMSD) contributes to neurodegenerative diseases like Alzheimer’s Disease (AD). This study examined REMSD’s effects on cognition, ER stress, and AD-like pathology in rats, alongside melatonin’s therapeutic potential. REMSD was induced for six days using the modified multiple platform method (MMPM). Cognitive function was tested via the Morris Water Maze (MWM). Rats received melatonin (20 mg/kg), 4-PBA (as a positive control, 100 mg/kg), or vehicle during deprivation/recovery periods. Western blotting and ELISA assessed ER stress markers (BiP, pIRE1, pPERK, peIF2-α, CHOP and GRP94, GADD34), Alzheimer related molecules (Aβ40/42, Tau, APP, GSK3β) and apoptosis-related proteins (Caspase 2, Caspase 12, BAX/Bcl-2). In addition, the mRNA expression levels of ATF4 and ATF6 were determined by PCR. Recovery sleep restored cognition, but melatonin/4-PBA enhanced it further. REMSD was associated with increased ER stress and AD-like pathology, whereas melatonin and 4-PBA appeared to attenuate these alterations, possibly by influencing the unfolded protein response (UPR) and reducing protein misfolding. Melatonin shows promise in countering SD-associated neurodegeneration, highlighting sleep’s role in proteostasis and its potential clinical use in AD and protein-misfolding disorders.

## Introduction

Sleep occupies one-third of our lives and is vital for both physiological and psychological functioning [1]. Studies show that 7–9 h of sleep per night is essential for cognitive function, memory consolidation, and overall health [2, 3]. Sleep deprivation (SD) has become more common due to lifestyle changes and can result from medical conditions, psychological stress, and environmental factors [4]. REM sleep deprivation (REMSD) has been associated with impaired health, increased apoptosis [5], and oxidative stress [6]. These disruptions may accelerate neurodegenerative processes, including Alzheimer’s disease (AD) [7].

AD, the most common form of dementia, is expected to increase in prevalence with an aging global population [8]. Despite extensive research, there is no definitive cure for AD, resulting in significant socioeconomic burdens [9]. SD has emerged as a potential risk factor for AD, exacerbating cognitive decline and pathological changes such as amyloid-β (Aβ) accumulation and tau hyperphosphorylation [10, 11], both key markers of AD. Chronic sleep disturbances may also impair glymphatic clearance, a system responsible for removing toxic protein aggregates from the brain during sleep [12, 13].

Sleep deprivation contributes to neurodegeneration through endoplasmic reticulum (ER) stress, disrupting proteostasis—the balance of protein folding and degradation. Under normal conditions, chaperones like ER chaperone glucose-regulated protein 78 (BiP/GRP78, Hspa5) ensure proper folding, but stress factors (e.g., oxidative stress, inflammation) overwhelm the ER, causing misfolded protein accumulation and triggering the unfolded protein response (UPR) [14]. The UPR is mediated by three primary sensors: inositol-requiring enzyme-1 (IRE1), protein kinase R-like ER kinase (PERK), and activating transcription factor 6 (ATF6) [15]. Upon ER stress, PERK phosphorylates eukaryotic initiation factor 2α (eIF-2α), halting protein synthesis while upregulating activating transcription factor 4 (ATF4). Chronic stress activates the pro-apoptotic ATF4-C/EBP homologous protein (CHOP) pathway [16, 17]. IRE1 splices X-box binding protein 1 (XBP1) mRNA to produce XBP1s, enhancing ER-associated degradation (ERAD) and chaperone production [18]. ATF6 is cleaved into ATF6f, upregulating ER chaperones and folding enzymes [19].

While the UPR initially restores homeostasis, persistent ER stress leads to cell death—a process implicated in diabetes, cancer, and neurodegenerative disorders [20]. In AD, postmortem studies reveal elevated ER stress markers, including phosphorylated PERK and eIF2α, in patient brain tissues [21]. Furthermore, ER stress contributes to tau pathology by inhibiting tau degradation and activating kinases like Glycogen Synthase Kinase 3-beta (GSK-3β) [22, 23].

SD contributes to ER stress, with increased levels of BiP, a marker of ER stress, observed after six or more hours of deprivation [24]. REMSD has been shown to induce apoptosis in various brain regions, likely due to unresolved protein misfolding and oxidative damage [25]. Studies have reported elevated ER stress markers in the brains of AD patients [26], further supporting the role of ER stress in AD progression.

Misfolded proteins within the ER activate the UPR, leading to an increase in endogenous chaperones. External administration of chemical chaperones like 4-phenylbutyric acid (4-PBA) has shown potential in reducing ER stress [27]. Melatonin, a hormone secreted by the pineal gland, regulates circadian rhythms and has neuroprotective, antioxidant, and anti-apoptotic properties [28]. Melatonin mitigates oxidative damage, reduces apoptosis in REMSD models, and enhances neurogenesis [29, 30]. Notably, melatonin levels decline with aging and AD progression, suggesting a protective role against neurodegeneration [31].

Despite established links between SD, ER stress, and AD, clinical applications remain underexplored. This study aims to investigate the effects of REMSD on cognitive function, ER stress signaling pathways, and AD-like pathology, while evaluating melatonin’s potential as a therapeutic intervention. Understanding these mechanisms may pave the way for novel treatments targeting sleep disturbances in neurodegenerative diseases.

## Methods

The study was approved by the Bursa Uludag University Local Ethics Committee on Animal Research under decision number 2020-13/04. Male Sprague Dawley rats, aged 8–12 weeks, were randomly assigned to groups (*n* = 9). Sample size was determined by a power analysis using G*Power, based on an α of 0.05 and a power of 0.80. Experiments were conducted in a room maintained at 22–24 °C with a 12-hour light/dark cycle. Based on previous studies demonstrating that apoptosis is induced by six days of REMSD [5, 32], this study included a 6-day REMSD period. REMSD was induced using the modified multiple platform method (MMPM), in which three animals were placed on six platforms inside a large tank for six consecutive days. Rats were placed on platforms (6.5 cm diameter) in a water tank (24 °C), where REM sleep was prevented by causing the animals to fall into the water when muscle tone was lost during REM sleep. For the animals in the environmental control (EC) group, the same-size tanks were used; however, grids were placed over the platforms to allow the animals to maintain normal REM sleep without experiencing SD. The dosage of melatonin (20 mg/kg) [32] and 4-PBA (used as a positive control and chaperone, 100 mg/kg) [33] were determined based on previous studies demonstrating their effectiveness. Both melatonin (Sigma-Aldrich, St. Louis, MO, USA) and 4-PBA (Sigma-Aldrich, St. Louis, MO, USA) were dissolved in dimethyl sulfoxide (DMSO, Sigma-Aldrich, St. Louis, MO, USA) and administered intraperitoneally (i.p.) at 08:00 a.m., coinciding with the end of the nocturnal cycle. The animals were randomly divided into the following groups:

### Naive Group

Animals in this group were housed under optimal conditions with ad libitum access to food and water in standard laboratory cages. On day 7, they were decapitated, and cortical and hippocampal tissues were collected.

### Study Group 1

These groups were designed to investigate the molecular changes occurring during REMSD and the effectiveness of the treatment. The animals were housed according to group-specific conditions (EC or REMSD) and received daily i.p. injections for six days. Decapitation and tissue collection were performed on day 7. Subgroups: EC + S, EC+DMSO, EC + PBA, EC + MEL, SD + S, SD+DMSO, SD + PBA, SD + MEL (all *n* = 9).

### Study Group 2

These groups were designed to investigate the molecular changes following REMSD, as well as the effectiveness of the treatment and sleep recovery (R). Animals were subjected to REMSD conditions for six days, followed by standard housing conditions for a recovery period (days 7–12), during which daily injections were administered. Animals were decapitated on day 13 for tissue collection. Subgroups: R-EC + S, R-EC+DMSO, R-EC + PBA, R-EC + MEL, R-SD + S, R-SD+DMSO, R-SD + PBA, R-SD + MEL (all *n* = 9).

### Behavioral Study (Morris Water Maze Test)

Rats underwent a 5-day Morris water maze test to evaluate the effects of REMSD and treatments on learning and memory. The tank (150 cm diameter, 60 cm height) was filled with black water (30 cm depth) at 22–24 °C. Trials were recorded and analyzed with EthoVision software.

During the 4-day training phase, rats were placed twice daily from different quadrants, allowed 90 s to find a hidden platform submerged 2 cm underwater below the water surface. Rats that found the platform stayed on it for 30 s; those that didn’t were guided to it. Escape latency was recorded each trial.

On day 5 (memory/probe phase), the platform was removed. Rats were placed opposite the former platform quadrant and allowed to swim for 90 s. Time spent in the target quadrant, latency to first platform area entry, and platform crossing frequency were recorded.

**Fig. 1:**
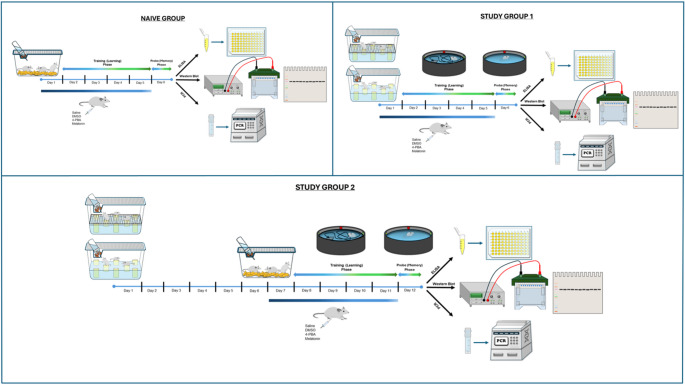
Experimental design and timeline

Study Group 1 trained on days 2–5 with memory tested on day 6; Study Group 2 trained on days 8–11 with memory tested on day 12 (Fig. 1).

### Western Blot Analyses

Animals were decapitated on day 7 or 13, and the right cortical and hippocampal tissues were dissected. The tissues were homogenized in cold Phosphate Buffered Saline (PBS, Sigma-Aldrich, St. Louis, MO, USA) with added Protease and Phosphatase Inhibitor Cocktail (Sigma-Aldrich, St. Louis, MO, USA) followed by centrifugation. The supernatants were transferred to microcentrifuge tubes and stored at −80 °C until analysis. Western blot analysis was performed to measure the levels of BiP (1:1000, Cell Signaling Technology, Danvers, MA, USA), pIRE1 (1:1000, St John’s Laboratory, London, UK), pPERK (1:1000, Cell Signaling Technology, Danvers, MA, USA), peIF2-α (1:1000, Cell Signaling Technology, Danvers, MA, USA) and CHOP (1:1000, Cell Signaling Technology, Danvers, MA, USA). Then, β-III-tubulin (1:1000, Cell Signaling Technology, Danvers, MA, USA), was analyzed as a structural protein to confirm equal protein loading. Western blot analyses were performed as described in a previous study [6].

### Enzyme-Linked Immunosorbent Assay (ELISA) Analyses

The collected supernatants mentioned in the Western blot section were also used for ELISA analyses. Aβ40, Aβ42, Tau, Amyloid Precursor Protein (APP), GSK3 β, Bcl-2, Bax, Caspase-2, Caspase-12, Glucose-regulated protein 94 (GRP94), GADD34 levels were analyzed in hippocampal and cortical tissues spectrophotometrically using commercial ELISA kits (BT-LAB, Shanghai Korain Biotech Co., Ltd., People’s Republic of China), following the manufacturer’s protocols.

### Real-Time Polymerase Chain Reaction (RT-PCR) Analyses

The dissected left cortical and hippocampal tissue samples were placed in RNAlater solution (GenemarkBio, Taiwan) to preserve RNA for extraction and stored at + 4 °C overnight. Nucleic acid isolation was performed using the Quick-DNA/RNA Miniprep Plus Kit (Zymo Research, Orange, CA), which enables simultaneous DNA and RNA isolation from tissue samples, following the manufacturer’s protocol. The purity and concentration of the obtained nucleic acids were measured using a NanoDrop™ 2000/2000c Spectrophotometer (Thermo Scientific). DNA samples were stored at −20 °C. Complementary DNA (cDNA) synthesis from the obtained RNA was performed using the GScript RTase kit (GeneDirex, Inc., USA). Specific primers for ATF4 and ATF6 were designed using the Primer3 software based on sequence information from the NCBI Gene database to assess gene expression levels. Gene expression levels were analyzed using the SimplyGreen qPCR Master Mix, No Rox kit (GeneDirex, Inc., USA) on a Bio-Rad CFX96 instrument (Bio-Rad, US). Following real-time PCR, melt curve analysis were conducted, and the resulting cycle threshold (Ct) values were normalized to a reference gene and evaluated using the 2^-ΔΔCt method.

### Statistical Analyses

Statistical analyses were performed using SigmaPlot version 12.5. The distribution characteristics of the variables were analyzed using the Shapiro-Wilk test, and homogeneity of variance was assessed using Levene’s test. Behavioral data from the Morris Water Maze training phase were analyzed using repeated-measures ANOVA, while probe trial performance, Western blot, ELISA, and PCR results were evaluated using one-way ANOVA. To control for multiple comparisons, p-values were adjusted using the Holm-Šidák correction method. All values are reported as mean ± SD. A p-value of less than 0.05 was considered statistically significant.

## Results

All comparisons were made relative to the saline groups, as no significant differences were observed between the saline- and DMSO-treated groups.

### Morris Water Maze Results

#### Training (Learning) Phase

In Study Group 1, homogeneity of variances was confirmed by Levene’s test (*p* > 0.05) for all analyses.

**Fig. 2:**
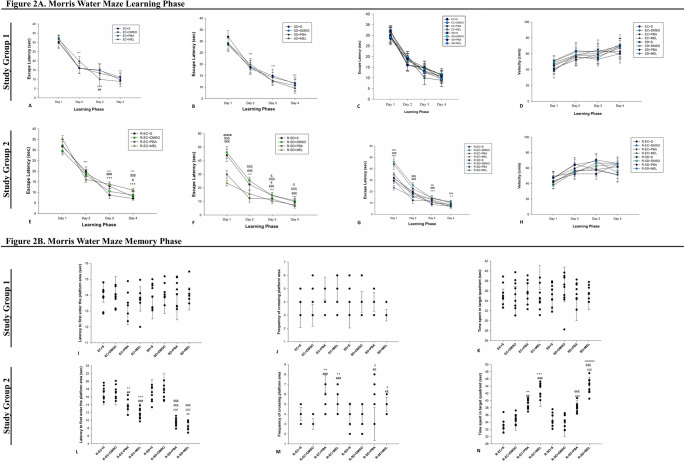
Morris water maze learning and memory phase results (*n* = 9). **A**–**D** Learning phase of study group 1, **E**–**H** Learning phase of study group 2, **I**–**K** Memory phase of study group 1, **L**–**N** Memory phase of study group 2. A single symbol indicates *p* < 0.05, two symbols *p* < 0.01, and three symbols *p* < 0.001. *Compared to Day 1 in all groups, ^+^Compared to R-EC + S or EC + S, ^#^Compared to EC+DMSO or R-EC+DMSO, ^$^Compared to R-EC + PBA, ^x^Compared to R-EC + MEL, ^ß^Compared to R-SD + S, ^€^Compared to R-SD+DMSO, ^æ^Compared to R-SD + PBA

During 4-day training period (η^2^ = 0.91), both EC and REMSD groups showed progressively shorter latencies vs. day 1 (*p* < 0.001). On day 3, EC + MEL had shorter latency than EC + S (*p* < 0.001). No significant differences were observed among REMSD groups. EC vs. REMSD groups receiving the same treatment showed no learning differences. No significant differences in locomotor activity (velocity) were observed among the groups on same testing day. Levene’s test confirmed homogeneity of variances (*p* > 0.05) and the effect size was η^2^ =0.73 (Fig. 2A-A, B, C, D).

In Study Group 2, homogeneity of variances was confirmed by Levene’s test (*p* > 0.05) for all analyses. During 4-day training period (η^2^ = 0.96), R-EC and R-SD groups showed improved performance over days (*p* < 0.001). On day 3, R-EC + PBA and R-EC + MEL showed longer latencies than R-EC + S (*p* < 0.001). On day 4, R-EC + MEL latency was longer than both R-EC + S and R-EC + PBA (*p* < 0.001). R-SD + PBA had shorter latencies than R-SD + S on all days (p-values from < 0.001 to < 0.05). R-SD + MEL showed even shorter latencies vs. R-SD + S throughout the training period (*p* < 0.001) and was faster than R-SD + PBA on day 1 (*p* < 0.001). Comparing R-EC and R-SD with same treatments, R-SD + S and R-SD + MEL groups took longer on days 1 and 3 (*p* < 0.001). R-SD + S and R-SD + PBA groups took longer on day 2 (*p* < 0.001). On day 4, R-SD + S and R-SD + MEL groups also had longer latencies than R-EC counterparts (*p* < 0.01, *p* < 0.001). No significant differences in locomotor activity (velocity) were observed among the groups on same testing day. Levene’s test confirmed homogeneity of variances (*p* > 0.05) and the effect size was η^2^ =0.65 (Fig. 2A–E, F, G, H).

#### Probe (Memory) Phase

In Study Group 1, homogeneity of variances was confirmed by Levene’s test (*p* > 0.05) for all analyses. No significant differences were observed in latency to first platform entry (η^2^ =0.13), platform crossings (η^2^ =0.11) or time spent in target quadrant (η^2^ =0.02) (Fig. 2B-I, J, K). In Study Group 2, homogeneity of variances was confirmed by Levene’s test (*p* > 0.05) for all analyses. Latency to first platform entry (η^2^ =0.84) was shorter in R-EC + PBA (*p* < 0.01) and R-EC + MEL (*p* < 0.001) vs. R-EC + S. Similarly, R-SD + PBA and R-SD + MEL had shorter latencies than R-SD + S (*p* < 0.001). R-SD + PBA and R-SD + MEL groups reached platform faster than their R-EC counterparts (*p* < 0.001, *p* < 0.01).

Platform crossing frequency (η^2^ =0.47) was higher in R-EC + PBA and R-EC + MEL vs. R-EC + S (*p* < 0.01, *p* < 0.001), and in R-SD + PBA and R-SD + MEL vs. R-SD + S (*p* < 0.05). No difference was found comparing same treatments between groups.

Time spent in target quadrant (η^2^ =0.85) was higher in R-EC + PBA (*p* < 0.01) and R-EC + MEL (*p* < 0.001) vs. R-EC + S; R-EC + MEL spent more time than R-EC + PBA (*p* < 0.001). Similarly, R-SD + PBA and R-SD + MEL spent more time than R-SD + S (*p* < 0.001) and R-SD + MEL spent more time than R-SD + PBA. No differences were observed between groups receiving same treatment (Fig. 2B-L, M, N).

### The Results of Western Blot Analyses

All Western blot expression data were normalized to the Naive group. Western blot analysis revealed significant group-dependent alterations in ER stress markers (BiP, pPERK, peIF2α, CHOP, and pIRE1) in the hippocampus and cortex tissues. The representative Western blot images are presented in Fig. 5.

#### Study Group 1 Western Blot Results

##### BiP Expression

**Fig. 3-4:**
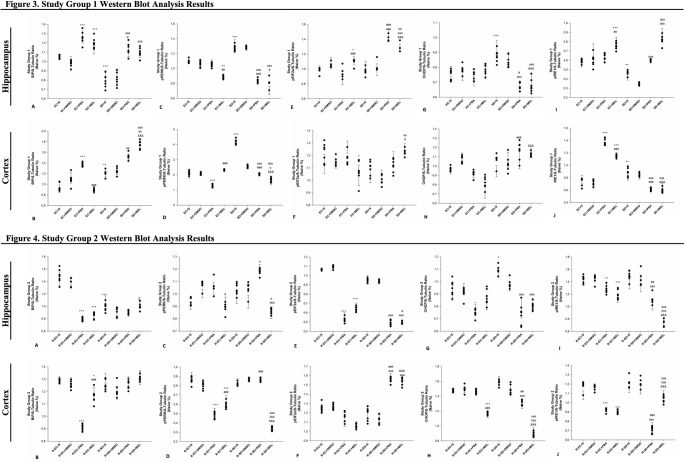
Western blot analysis results for study group 1 (Fig. 3) and study group 2 (Fig. 4). **A**-**C**-**E**-**G**-**I** hippocampus, **B**-**D**-**F**-**H**-**J** cortex results (*n* = 7). A single symbol indicates *p* < 0.05, two symbols *p* < 0.01, three symbols *p* < 0.001. ^+^ Compared to EC + S or R-EC + S, ^&^ Compared to EC + MEL or R-EC + MEL, ^#^ Compared to EC + PBA or R-EC + PBA, ^a^Compared to SD + S or R-SD + S, ^b^Compared to SD + PBA or R-SD + PBA

In the hippocampus, Levene’s test confirmed homogeneity of variances (*p* > 0.05) and the effect size was η^2^ = 0.90. BiP levels significantly increased in EC + PBA (*p* < 0.001) and EC + MEL (*p* < 0.001) compared to EC + S. Similarly, SD + PBA (*p* < 0.001) and SD + MEL (*p* < 0.001) groups exhibited elevated BiP expression relative to SD + S. Notably, SD + S showed significantly lower BiP expression than EC + S (*p* < 0.001). SD + PBA showed lower BiP levels than EC + PBA (*p* < 0.05). In the cortex, Levene’s test confirmed homogeneity of variances (*p* > 0.05) and the effect size was η^2^ = 0.95. BiP levels were elevated in EC + PBA vs. EC + S (*p* < 0.001). However, EC + MEL showed reduced expression of BiP compared to EC + PBA (*p* < 0.001) and SD + MEL (*p* < 0.001). SD + S exhibited significantly higher cortical BiP than EC + S (*p* < 0.01). BiP levels were also higher in SD + PBA (*p* < 0.01) and SD + MEL (*p* < 0.001) vs. SD + S. SD + MEL showed significantly higher BiP expression than SD + PBA (*p* < 0.01) (Fig. 3A, B).

##### pPERK Expression

In the hippocampus, Levene’s test confirmed homogeneity of variances (*p* > 0.05) and the effect size was η^2^ = 0.97. Hippocampal pPERK was significantly reduced in EC + MEL compared to EC + S (*p* < 0.01) and EC + PBA (*p* < 0.01). SD + PBA and SD + MEL groups also showed decreased levels of pPERK vs. SD + S (*p* < 0.001). SD + S rats had higher pPERK than EC + S (*p* < 0.001), while SD + PBA had lower expression than EC + PBA (*p* < 0.001). SD + MEL showed significantly lower pPERK expression than EC + MEL (*p* < 0.05). In the cortex, Levene’s test confirmed homogeneity of variances (*p* > 0.05) and the effect size was η^2^ = 0.98. EC + PBA showed lower levels of pPERK levels than EC + S and EC + MEL (*p* < 0.001). SD + S rats exhibited significantly higher cortical pPERK than EC + S (*p* < 0.001), but both SD + PBA and SD + MEL showed reductions vs. SD + S (*p* < 0.001). SD + PBA had higher expression than EC + PBA (*p* < 0.001), and SD + MEL was lower than EC + MEL (*p* < 0.001) and SD + PBA (*p* < 0.05) (Fig. 3C, D).

##### peIF2α Expression

In the hippocampus, Levene’s test confirmed homogeneity of variances (*p* > 0.05) and the effect size was η^2^ = 0.90. EC + MEL exhibited higher peIF2α levels than EC + S (*p* < 0.05) and EC + PBA (*p* < 0.001).

Both SD + PBA and SD + MEL groups showed elevated expression of peIF2α compared to SD + S (*p* < 0.001), but SD + MEL had significantly lower levels of peIF2α than SD + PBA (*p* < 0.001). SD + PBA had higher expression of peIF2α than EC + PBA (*p* < 0.001), and SD + MEL had higher expression of peIF2α than EC + MEL (*p* < 0.001). In the cortex, Levene’s test confirmed homogeneity of variances (*p* > 0.05) and the effect size was η^2^ = 0.65. EC + MEL showed lower peIF2α levels than EC + S (*p* < 0.05), while SD + MEL had higher expression than both SD + S (*p* < 0.01) and EC + MEL (*p* < 0.05) (Fig. 3E, F).

##### CHOP Expression

In the hippocampus, Levene’s test confirmed homogeneity of variances (*p* > 0.05) and the effect size was η^2^ = 0.81. No significant differences were found among EC groups in hippocampal CHOP levels. In contrast, SD + PBA and SD + MEL groups had significantly reduced CHOP expression vs. SD + S (*p* < 0.001). SD + S showed increased CHOP levels vs. EC + S (*p* < 0.001). SD + PBA had lower levels of CHOP than EC + PBA (*p* < 0.05), and SD + MEL was lower than EC + MEL (*p* < 0.001). In the cortex, Levene’s test confirmed homogeneity of variances (*p* > 0.05), and the effect size was η^2^ = 0.79. SD + PBA and SD + MEL had higher CHOP levels than EC + PBA and EC + MEL, respectively (*p* < 0.001) (Fig. 3G, H).

##### pIRE1 Expression

In the hippocampus, Levene’s test confirmed homogeneity of variances (*p* > 0.05) and the effect size was η^2^ = 0.94. Hippocampal pIRE1 expression was elevated in EC + MEL compared to EC + S (*p* < 0.001) and EC + PBA (*p* < 0.01). SD + PBA and SD + MEL showed higher levels than SD + S (*p* < 0.001), and SD + MEL exceeded SD + PBA. Additionally, SD + S was significantly lower than EC + S (*p* < 0.01). In the cortex, Levene’s test confirmed homogeneity of variances (*p* > 0.05) and the effect size was η^2^ = 0.97. EC + PBA, EC + MEL (both *p* < 0.001), and SD + S (*p* < 0.01) groups had higher pIRE1 than EC + S. However, SD + PBA and SD + MEL had reduced levels compared to SD + S (*p* < 0.001). SD + PBA was lower expression of pIRE1 than EC + PBA (*p* < 0.001), and SD + MEL was lower than both EC + MEL and SD + PBA (*p* < 0.001) (Fig. 3I, J).

#### Study Group 2 Western Blot Results

##### BiP Expression

In the hippocampus, Levene’s test confirmed homogeneity of variances (*p* > 0.05) and the effect size was η^2^ = 0.94. Hippocampal BiP expression was significantly decreased in R-EC + PBA, R-EC + MEL, and R-SD + S compared to R-EC + S (*p* < 0.001), while R-SD + MEL had higher expression than R-EC + MEL (*p* < 0.05). In the cortex, Levene’s test confirmed homogeneity of variances (*p* > 0.05), and the effect size was η^2^ = 0.93. BiP expression was lower in R-EC + PBA (*p* < 0.001) and R-EC + MEL (*p* < 0.05) vs. R-EC + S, but R-EC + MEL showed higher levels than R-EC + PBA (*p* < 0.001). R-SD + MEL also showed elevated BiP expression compared to R-EC + MEL (*p* < 0.05) (Fig. 4A, B).

##### pPERK Expression

In the hippocampus, Levene’s test confirmed homogeneity of variances (*p* > 0.05) and the effect size was η^2^ = 0.88. pPERK expression was reduced in R-EC + MEL vs. both R-EC + S and R-EC + PBA (*p* < 0.05), and in R-SD + MEL vs. R-SD + S (*p* < 0.05) and R-SD + PBA (*p* < 0.001). pPERK levels were increased in R-SD + PBA vs. R-SD + S (*p* < 0.05). In the cortex, Levene’s test confirmed homogeneity of variances (*p* > 0.05) and the effect size was η^2^ = 0.98. Cortical pPERK expression was lower in both R-EC + PBA and R-EC + MEL compared to R-EC + S (*p* < 0.001), though higher in R-EC + MEL than R-EC + PBA (*p* < 0.001). R-SD + MEL showed reduced cortical pPERK vs. both R-SD + S, R-SD + PBA (*p* < 0.001) and R-EC + MEL (*p* < 0.001). pPERK expression increased in R-SD + PBA compared to R-EC + PBA (*p* < 0.001) (Fig. 4C, D).

##### peIF2α Expression

In the hippocampus, Levene’s test confirmed homogeneity of variances (*p* > 0.05) and the effect size was η^2^ = 0.99. peIF2α was significantly reduced in R-EC + PBA, R-EC + MEL, R-SD + PBA, and R-SD + MEL compared to their respective saline groups (*p* < 0.001). R-SD + MEL was also lower than R-EC + MEL (*p* < 0.05). In the cortex, Levene’s test confirmed homogeneity of variances (*p* > 0.05) and the effect size was η^2^ = 0.95. peIF2α was elevated in R-SD + PBA and R-SD + MEL compared to R-SD + S (*p* < 0.001). Additionally, both groups showed higher levels than their EC-treated counterparts (*p* < 0.001 and *p* < 0.001, respectively) (Fig. 4E, F).

##### CHOP Expression

In the hippocampus, Levene’s test confirmed homogeneity of variances (*p* > 0.05) and the effect size was η^2^ = 0.85. Hippocampal CHOP levels were reduced in R-EC + PBA vs. R-EC + S (*p* < 0.05), and in both R-SD + PBA and R-SD + MEL vs. R-SD + S (*p* < 0.001). In the cortex, Levene’s test confirmed homogeneity of variances (*p* > 0.05), and the effect size was η^2^ = 0.97. Cortical CHOP expression was lowest in R-EC + MEL compared to both R-EC + S and R-EC + PBA (*p* < 0.001). R-SD + PBA and R-SD + MEL had lower levels than R-SD + S (*p* < 0.001), with R-SD + MEL being significantly lower than R-SD + PBA (*p* < 0.001). Both were also significantly lower than the corresponding EC-treated groups (*p* < 0.01 and *p* < 0.001, respectively) (Fig. 4G, H).

##### pIRE1 Expression

In the hippocampus, Levene’s test confirmed homogeneity of variances (*p* > 0.05) and the effect size was η^2^ = 0.92. Hippocampal pIRE1 expression was reduced in R-EC + PBA (*p* < 0.01), R-EC + MEL (*p* < 0.001), R-SD + PBA and R-SD + MEL (both *p* < 0.001) vs. their respective saline controls. R-SD + MEL was also lower than R-SD + PBA (*p* < 0.001), and both were lower than EC groups receiving the same treatments (*p* < 0.01 and *p* < 0.001).

**Fig. 5:**
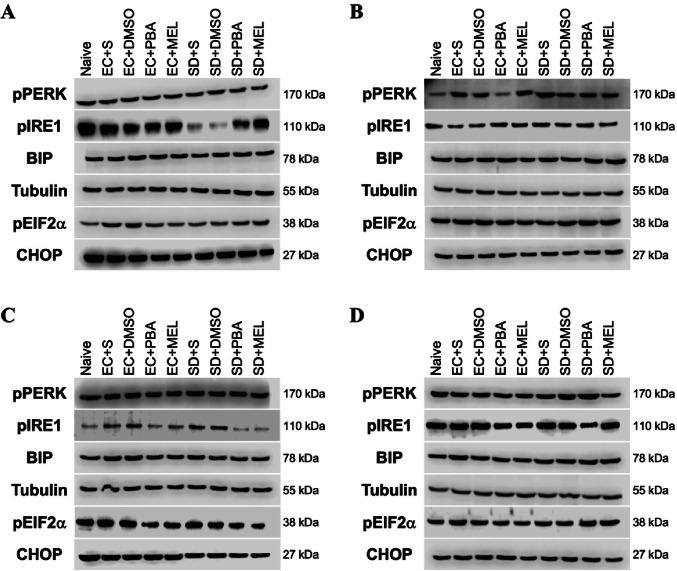
Representative western blot images. **A** Study group 1 hippocampus, **B** study group 1 cortex, **C** study group 2 hippocampus, **D** study group 2 cortex

In the cortex, Levene’s test confirmed homogeneity of variances (*p* > 0.05), and the effect size was η^2^ = 0.96. pIRE1 expression was lower in R-EC + PBA and R-EC + MEL vs. R-EC + S (*p* < 0.001). R-SD + PBA showed reduced levels vs. R-SD + S (*p* < 0.001), but higher than R-SD + MEL (*p* < 0.001) and lower than R-EC + PBA (*p* < 0.001). R-SD + PBA also showed reduced levels vs. R-SD + S (*p* < 0.001). R-SD + MEL showed increased cortical pIRE1 levels compared to R-EC + MEL (*p* < 0.001) (Figs. 4I, J; 5).

### The Results of ELISA Analysis

**Fig. 6:**
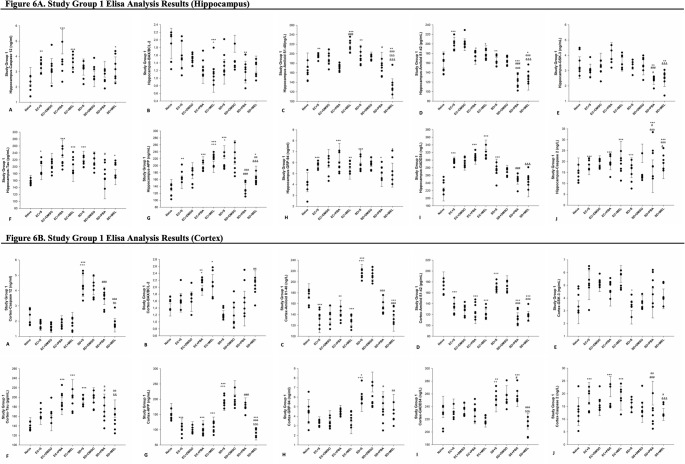
ELISA analyses of hippocampus (**A**) and cortex samples (**B**) from study group 1 (*n* = 7). A single symbol indicates *p* < 0.05, two symbols *p* < 0.01, three symbols *p* < 0.001. *Compared to naive group; ^+^compared to EC + S; ^#^compared to EC + PBA; ^&^compared to EC + MEL; ^a^compared to SD + S; ^b^compared to SD + PBA

**Fig. 7:**
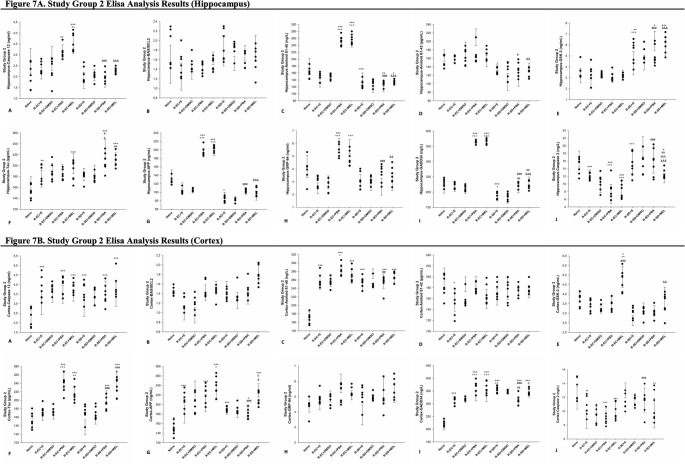
ELISA analyses of hippocampus (**A**) and cortex samples (**B**) from study group 2 (*n* = 7). A single symbol indicates *p* < 0.05, two symbols *p* < 0.01, three symbols *p* < 0.001. *Compared to naive group; ^+^compared to R-EC + S; ^#^compared to R-EC + PBA; ^&^compared to R-EC + MEL; ^a^compared to R-SD + S; ^b^compared to R-SD + PBA

To investigate the role of SD in ER stress, Alzheimer-like pathology, and the apoptotic process, as well as the effect of melatonin treatment on these events, several parameters involved in these processes were examined using the ELISA method. Since no significant differences were observed between the rats treated with saline and those treated with DMSO, comparisons were made only within the saline groups. The results are presented under three main headings: ER stress, apoptosis and Alzheimer-like pathology-related parameters (Figs. 6 and 7).

#### ER Stress-Related Parameters

To evaluate the impact of sleep deprivation and melatonin treatment on ER stress, GRP94 and GADD34 levels were measured via ELISA in hippocampal and cortical tissues of Study Groups 1 and 2.

##### GRP94 Expression

In the hippocampus of Study Group 1, Levene’s test confirmed homogeneity of variances (*p* > 0.05) and the effect size was η^2^ = 0.43. Hippocampal GRP94 levels were significantly elevated in all experimental groups (EC + S, EC + PBA, EC + MEL, SD + S, SD + PBA, SD + MEL) compared to the Naive group (*p* < 0.05 to *p* < 0.001). In the cortex, Levene’s test confirmed homogeneity of variances (*p* > 0.05), and the effect size was η^2^ = 0.60. SD + S showed increased levels of GRP94 compared to both the Naive (*p* < 0.05) and EC + S (*p* < 0.001) groups. However, GRP94 levels were reduced in SD + PBA (*p* < 0.05) and SD + MEL (*p* < 0.01) compared to SD + S.

In the hippocampus of Study Group 2, Levene’s test confirmed homogeneity of variances (*p* > 0.05) and the effect size was η^2^ = 0.71. Hippocampal GRP94 was elevated in R-EC + PBA vs. Naive (*p* < 0.001), and in R-EC + PBA and R-EC + MEL vs. R-EC + S (*p* < 0.001). However, SD treatments (R-SD + PBA, R-SD + MEL) significantly reduced hippocampal GRP94 compared to EC counterparts (*p* < 0.01 to *p* < 0.001). In the cortex, Levene’s test confirmed homogeneity of variances (*p* > 0.05), and the effect size was η^2^ = 0.31. No significant differences were observed in cortical GRP94 levels among the groups.

##### GADD34 Expression

In the hippocampus of Study Group 1, Levene’s test confirmed homogeneity of variances (*p* > 0.05) and the effect size was η^2^ = 0.83. Hippocampal GADD34 levels were higher in EC + S, EC + PBA, EC + MEL, and SD + S vs. Naive (*p* < 0.01 to *p* < 0.001). SD + PBA and SD + MEL groups showed reduced hippocampal GADD34 compared to corresponding EC groups (*p* < 0.001). In the cortex, Levene’s test confirmed homogeneity of variances (*p* > 0.05) and the effect size was η^2^ = 0.69. Cortically, GADD34 was elevated in SD + S vs. Naive (*p* < 0.01) and EC + S (*p* < 0.01), and also in SD + PBA vs. Naive (*p* < 0.001). SD + MEL showed decreased levels of GADD34 vs. both SD + S and SD + PBA (*p* < 0.001).

In the hippocampus of Study Group 2, Levene’s test confirmed homogeneity of variances (*p* > 0.05) and the effect size was η^2^ = 0.98. Hippocampal GADD34 was higher in R-EC + PBA and R-EC + MEL vs. Naive and R-EC + S (*p* < 0.001) but reduced in R-SD + S vs. Naive and R-EC + S (*p* < 0.001 and *p* < 0.05, respectively). Both R-SD + PBA and R-SD + MEL had higher levels than R-SD + S (*p* < 0.01), yet still significantly lower than R-EC groups receiving the same treatment (*p* < 0.001). In the cortex, Levene’s test confirmed homogeneity of variances (*p* > 0.05) and the effect size was η^2^ = 0.90. All treatment groups showed higher GADD34 levels compared to Naive (*p* < 0.001). GADD34 levels were also elevated in R-EC + PBA, R-EC + MEL, and R-SD + S vs. R-EC + S (*p* < 0.001 to *p* < 0.01). R-SD + PBA had lower levels than R-SD + S (*p* < 0.01) and R-EC + PBA (*p* < 0.001), while R-SD + MEL had higher levels than R-SD + PBA (*p* < 0.05).

#### Apoptosis-Related Parameters

To evaluate the role of sleep deprivation in apoptosis and the potential protective effects of melatonin, levels of Caspase-12, Bcl-2 associated X protein (Bax), B-cell lymphoma 2 (Bcl-2), and Caspase-2 were measured by ELISA in homogenates from the hippocampus and cortex of all rats in Study Groups 1 and 2.

##### Caspase-12 Analysis

In the hippocampus of Study Group 1, Levene’s test confirmed homogeneity of variances (*p* > 0.05) and the effect size was η^2^ = 0.42. Hippocampal Caspase-12 levels were significantly elevated in the EC + S (*p* < 0.01), EC + PBA (*p* < 0.001), EC + MEL (*p* < 0.001), and SD + MEL (*p* < 0.05) groups compared to the Naive group. In the cortex, Levene’s test confirmed homogeneity of variances (*p* > 0.05) and the effect size was η^2^ = 0.85. Caspase-12 levels were significantly higher in the SD + S group compared to the Naive (*p* < 0.001) and EC + S (*p* < 0.001) groups. Additionally, levels were higher in the SD + PBA group compared to the EC + PBA group (*p* < 0.001). However, Caspase-12 levels were significantly reduced in the SD + MEL group compared to the SD + S group (*p* < 0.001) and SD + PBA (*p* < 0.01).

In the hippocampus of Study Group 2, Levene’s test confirmed homogeneity of variances (*p* > 0.05) and the effect size was η^2^ = 0.69. Hippocampal Caspase-12 levels were significantly increased in the R-EC + PBA (*p* < 0.01) and R-EC + MEL (*p* < 0.001) groups compared to the Naive group. Conversely, levels were significantly decreased in the R-SD + PBA and R-SD + MEL groups compared to their respective EC counterparts (*p* < 0.001 for both). In the cortex, Levene’s test confirmed homogeneity of variances (*p* > 0.05) and the effect size was η^2^ = 0.52. Caspase-12 levels were significantly higher in all experimental groups (R-EC + S, R-EC + PBA, R-EC + MEL, R-SD + S, R-SD + PBA, and R-SD + MEL; all *p* < 0.001) compared to the Naive group.

##### BAX/BCL-2 Analysis

In the hippocampus of Study Group 1, Levene’s test confirmed homogeneity of variances (*p* > 0.05) and the effect size was η^2^ = 0.34. Bax/Bcl-2 ratio was significantly lower in the EC + MEL (*p* < 0.001) and SD + PBA (*p* < 0.01) groups compared to the Naive group. Additionally, the EC + MEL group showed a significantly lower ratio than the EC + S group (*p* < 0.05). In the cortex, Levene’s test confirmed homogeneity of variances (*p* > 0.05) and the effect size was η^2^ = 0.56. Bax/Bcl-2 ratio was significantly higher in the EC + PBA (*p* < 0.01) and EC + MEL (*p* < 0.05) groups compared to the Naive group. Furthermore, the ratio was higher in the EC + PBA group compared to the EC + S group (*p* < 0.05), and in the SD + MEL group compared to the SD + S group (*p* < 0.01). In the Study Group 2, Levene’s test confirmed homogeneity of variances (*p* > 0.05) and the effect size was η^2^ = 0.34 for hippocampus and η^2^ = 0.58 for cortex. No significant differences in Bax/Bcl-2 ratio were observed among the groups.

##### Caspase-2 Analysis

In the hippocampus of Study Group 1, Levene’s test confirmed homogeneity of variances (*p* > 0.05) and the effect size was η^2^ = 0.29. Caspase-2 levels were significantly elevated in EC + S, EC + PBA, EC + MEL, SD + PBA, and SD + MEL groups compared to Naive (all *p* < 0.001). Levels of Caspase-2 in SD + S were lower than EC + S (*p* < 0.001), but SD + PBA and SD + MEL were higher than SD + S (both *p* < 0.001). Caspase-2 levels were also higher in SD + PBA vs. EC + PBA (*p* < 0.05) and SD + MEL vs. EC + MEL (*p* < 0.05). In the cortex, Levene’s test confirmed homogeneity of variances (*p* > 0.05), and the effect size was η^2^ = 0.24. Caspase-2 levels were increased in EC + S, EC + PBA, and EC + MEL vs. Naive (all *p* < 0.001). Among SD groups, Caspase-2 levels were lower in SD + PBA (*p* < 0.01) and SD + MEL (*p* < 0.05) vs. SD + S. Levels in SD + PBA and SD + MEL were also lower than EC + PBA and EC + MEL respectively (both *p* < 0.001).

In the hippocampus of Study Group 2, Levene’s test confirmed homogeneity of variances (*p* > 0.05) and the effect size was η^2^ = 0.61. Caspase-2 levels decreased in R-EC + S, R-EC + PBA, R-EC + MEL (all *p* < 0.001), and R-SD + MEL (*p* < 0.05) vs. Naive. R-EC + PBA and R-EC + MEL were lower than R-EC + S (*p* < 0.001). Caspase-2 was higher in R-SD + S vs. R-EC + S, R-SD + PBA vs. R-EC + PBA, and R-SD + MEL vs. R-EC + MEL (all *p* < 0.001). Notably, R-SD + MEL was lower than R-SD + PBA (*p* < 0.001) and R-SD + S (*p* < 0.01). In the cortex, Levene’s test confirmed homogeneity of variances (*p* > 0.05) and the effect size was η^2^ = 0.45. Caspase-2 decreased in R-EC + S (*p* < 0.01), R-EC + PBA, R-EC + MEL, and R-SD + MEL (all *p* < 0.001) vs. Naive, but was higher in R-SD + PBA compared to R-EC + PBA (*p* < 0.001).

#### Alzheimer’s-Like Pathology Related Parameters

To investigate the role of SD in the development of Alzheimer-like pathology and the effects of melatonin treatment, the levels of Ab1-40, Ab1-42, GSK-3, Tau, and APP were measured using the ELISA method in hippocampal and cortical tissue homogenates from all rats in Study Group 1 and Study Group 2.

##### Aβ1–40

In the hippocampus of Study Group 1, Levene’s test confirmed homogeneity of variances (*p* > 0.05) and the effect size was η^2^ = 0.78. Hippocampal Aβ1–40 levels were significantly elevated in EC + S (*p* < 0.01), EC + MEL (*p* < 0.001) and SD + S (*p* < 0.01) and decreased in SD + MEL (*p* < 0.01) groups vs. Naive. EC + MEL group showed higher levels of Aβ1–40 than EC + PBA (*p* < 0.001), while SD + MEL had lower levels than EC + MEL (*p* < 0.001), SD + S (*p* < 0.001) and SD + PBA (*p* < 0.001). In addition, SD + PBA had lower levels of Aβ1–40 than SD + S (*p* < 0.05). In the cortex, Levene’s test confirmed homogeneity of variances (*p* > 0.05), and the effect size was η^2^ = 0.88. SD + S group had significantly higher levels of Aβ1–40 than Naive (*p* < 0.001). EC + S (*p* < 0.001), EC + PBA (*p* < 0.01), EC + MEL (*p* < 0.001) and SD + MEL (*p* < 0.001) showed reductions and both SD + PBA and SD + MEL were significantly lower than SD + S (*p* < 0.001).

In the hippocampus of Study Group 2, Levene’s test confirmed homogeneity of variances (*p* > 0.05) and the effect size was η^2^ = 0.96. Hippocampal Aβ1–40 was significantly reduced in R-SD + S (*p* < 0.001), R-SD + PBA (*p* < 0.01), and R-SD + MEL (*p* < 0.05) vs. Naive, but increased in R-EC + PBA and R-EC + MEL (*p* < 0.001). Both R-SD + PBA and R-SD + MEL were significantly lower than R-EC + PBA and R-EC + MEL (*p* < 0.001). In the cortex, Levene’s test confirmed homogeneity of variances (*p* > 0.05), and the effect size was η^2^ = 0.86. All experimental groups (R-EC and R-SD subgroups) had higher Aβ1–40 levels vs. Naive. R-EC + PBA had significantly higher levels of Aβ1–40 than R-EC + S (*p* < 0.05), while R-SD + PBA was lower than R-EC + PBA (*p* < 0.01).

##### Aβ1–42

In the hippocampus of Study Group 1, Levene’s test confirmed homogeneity of variances (*p* > 0.05) and the effect size was η^2^ = 0.85. Hippocampal Aβ1–42 levels were elevated in EC + S (*p* < 0.001), EC + PBA and EC + MEL (*p* < 0.05) versus Naive, while they were reduced in SD + PBA (*p* < 0.001) and SD + MEL (*p* < 0.05). Aβ1–42 levels in SD + S were significantly lower than those in EC + S (*p* < 0.01). SD + PBA and SD + MEL both had lower levels of Aβ1–42 levels than EC + PBA, EC + MEL, and SD + S (all *p* < 0.001). In the cortex, Levene’s test confirmed homogeneity of variances (*p* > 0.05), and the effect size was η^2^ = 0.86. Aβ1–42 levels were decreased in EC and SD treatment groups (except SD + S) vs. Naive (*p* < 0.001). SD + S had higher levels of Aβ1–42 than EC + S (*p* < 0.001) and was reduced by both PBA and MEL treatments (*p* < 0.001).

In the hippocampus of Study Group 2, Levene’s test confirmed homogeneity of variances (*p* > 0.05) and the effect size was η^2^ = 0.61. Hippocampal Aβ1–42 levels were significantly lower in R-SD + MEL compared to R-EC + MEL (*p* < 0.01), R-SD + S compared to R-EC + S (*p* < 0.05) and R-SD + PBA (*p* < 0.05) compared to R-EC + PBA. In the cortex, Levene’s test confirmed homogeneity of variances (*p* > 0.05), and the effect size was η^2^ = 0.33. EC + S group had significantly lower levels of Aβ1–42 than Naive (*p* < 0.05).

##### GSK-3

In the hippocampus of Study Group 1, Levene’s test confirmed homogeneity of variances (*p* > 0.05), and the effect size was η^2^ = 0.51. Hippocampal GSK-3 levels were decreased in SD + PBA and SD + MEL compared to Naive (*p* < 0.01) and further reduced versus EC + PBA (*p* < 0.001) and EC + MEL (*p* < 0.001). In addition, SD + PBA had lower levels of GSK-3 than SD + S (*p* < 0.05). In the cortex, Levene’s test confirmed homogeneity of variances (*p* > 0.05), and the effect size was η^2^ = 0.50. SD + S showed lower levels of GSK-3 than EC + S (*p* < 0.05); no other significant differences were found.

In the hippocampus of Study Group 2, Levene’s test confirmed homogeneity of variances (*p* > 0.05), and the effect size was η^2^ = 0.74. Hippocampal GSK-3 levels were elevated in R-SD + S (*p* < 0.01), R-SD + PBA (*p* < 0.05), and R-SD + MEL (*p* < 0.001) versus Naive. These were also higher than respective EC groups (*p* < 0.001). In the cortex, Levene’s test confirmed homogeneity of variances (*p* > 0.05), and the effect size was η^2^ = 0.68. R-EC + MEL showed increased levels of GSK-3 compared to Naive (*p* < 0.01), R-EC + S (*p* < 0.05) and R-EC + PBA (*p* < 0.001), while R-SD + MEL was reduced versus R-EC + MEL (*p* < 0.01).

##### Tau

In the hippocampus of Study Group 1, Levene’s test confirmed homogeneity of variances (*p* > 0.05), and the effect size was η^2^ = 0.50. Hippocampal tau was elevated in EC + S (*p* < 0.05), EC + PBA, EC + MEL, SD + S (*p* < 0.001), and SD + MEL (*p* < 0.05) compared to Naive. SD + PBA had lower levels of tau than EC + PBA (*p* < 0.05). In the cortex, Levene’s test confirmed homogeneity of variances (*p* > 0.05), and the effect size was η^2^ = 0.61. Tau levels were significantly higher in EC + PBA, EC + MEL, and SD + S (*p* < 0.001) compared to naive and reduced in SD + MEL (*p* < 0.01) and SD + PBA (*p* < 0.05) versus SD + S. Tau levels were lower in SD + PBA and SD + MEL compared to EC + PBA and EC + MEL (*p* < 0.05 and *p* < 0.01).

In the hippocampus of Study Group 2, Levene’s test confirmed homogeneity of variances (*p* > 0.05), and the effect size was η^2^ = 0.65. Hippocampal tau levels increased in all R-EC and R-SD groups (except R-SD + S) versus Naive (*p* < 0.05–0.001). R-SD + PBA and R-SD + MEL had significantly higher levels of tau than R-SD + S (*p* < 0.05, *p* < 0.01) and tau levels increased in R-SD + PBA compared to R-EC + PBA (*p* < 0.05). In the cortex, Levene’s test confirmed homogeneity of variances (*p* > 0.05), and the effect size was η^2^ = 0.76. Tau levels were significantly elevated in R-EC + PBA, R-EC + MEL, R-SD + PBA, and R-SD + MEL versus Naive (*p* < 0.001). Tau levels were higher in R-EC + PBA and R-EC + MEL compared to R-EC + S (*p* < 0.001, *p* < 0.01). Levels of Tau in R-SD + MEL were higher than R-SD + S and R-SD + PBA (*p* < 0.001, *p* < 0.05), while they were lower in R-SD + PBA was lower than R-EC + PBA (*p* < 0.001).

##### APP

In the hippocampus of Study Group 1, Levene’s test confirmed homogeneity of variances (*p* > 0.05), and the effect size was η^2^ = 0.76. Hippocampal APP levels increased in EC + S (*p* < 0.01), SD + MEL (*p* < 0.05), EC + PBA, EC + MEL, and SD + S (*p* < 0.001) vs. Naive. APP levels in EC + MEL were higher than EC + S (*p* < 0.001). SD + S had higher APP levels compared to EC + S (*p* < 0.05). APP was reduced in SD + PBA (*p* < 0.001) and SD + MEL (*p* < 0.01) vs. SD + S, and also lower than EC + PBA and EC + MEL (*p* < 0.001). In the cortex, Levene’s test confirmed homogeneity of variances (*p* > 0.05), and the effect size was η^2^ = 0.89. EC + S, EC + PBA, EC + MEL, and SD + MEL showed reduced levels of APP vs. Naive (*p* < 0.001), whereas SD + S had elevated levels of APP (*p* < 0.001). SD + S also showed higher levels of APP than EC + S (*p* < 0.001). SD + MEL had lower levels of APP compared to SD + S and SD + PBA groups (*p* < 0.001), with SD + PBA higher levels of APP than EC + PBA (*p* < 0.001).

In the hippocampus of Study Group 2, Levene’s test confirmed homogeneity of variances (*p* > 0.05), and the effect size was η^2^ = 0.98. Hippocampal APP levels increased in R-EC + PBA and R-EC + MEL (*p* < 0.001) but decreased in R-SD + S (*p* < 0.05) vs. Naive. R-EC + PBA and R-EC + MEL had higher levels of APP than R-EC + S (*p* < 0.001), while R-SD + PBA and R-SD + MEL had lower levels of APP than R-SD + S (*p* < 0.001). In the cortex, Levene’s test confirmed homogeneity of variances (*p* > 0.05), and the effect size was η^2^ = 0.76. All R-EC and R-SD groups had elevated levels of APP vs. Naive (*p* < 0.001, *p* < 0.05). R-EC + MEL had higher levels of APP than R-EC + S (*p* < 0.05), and R-SD + PBA and R-SD + MEL had lower levels of APP than EC groups (*p* < 0.001, *p* < 0.05). R-SD + MEL had significantly higher levels of APP than R-SD + PBA (*p* < 0.05).

### The Results of PCR Analysis

To investigate the role of SD in ER stress and the effects of melatonin treatment, key parameters involved in these processes were examined using the PCR method. Since no significant difference was observed between saline and DMSO- treated rats, comparisons were made only based on the saline groups. The PCR analysis results of the hippocampal and cortical tissues from the Naive Group, Study Group 1, and Study Group 2 are presented in Table 1A and B.

**Table 1:**
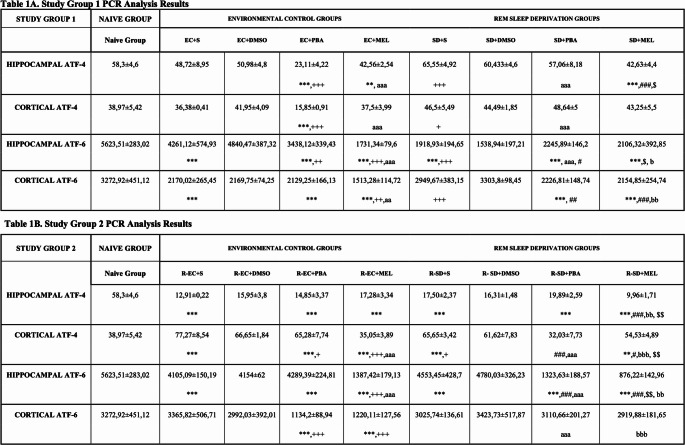
**A** PCR analysis results of study group 1 (*n* = 7). **B** PCR analysis results of study group 2 (*n* = 7)

#### ATF4

In the hippocampus of Study Group 1, Levene’s test confirmed homogeneity of variances (*p* > 0.05), and the effect size was η^2^ = 0.92. Hippocampal ATF4 levels were significantly reduced in EC + PBA (*p* < 0.001), EC + MEL (*p* < 0.01), and SD + MEL (*p* < 0.01) vs. Naive. EC + PBA had lower levels of ATF4 than EC + S and EC + MEL (*p* < 0.001). SD + MEL had lower levels of ATF4 than SD + S (*p* < 0.001) and SD + PBA (*p* < 0.05); SD + PBA was higher than EC + PBA (*p* < 0.001). In the cortex, Levene’s test confirmed homogeneity of variances (*p* > 0.05), and the effect size was η^2^ = 0.93. EC + PBA had lower levels of ATF4 than Naive and EC + S (*p* < 0.001). EC + MEL and SD + PBA had higher levels of ATF4 than EC + PBA (*p* < 0.001). ATF4 levels were significantly higher in SD + S group compared to EC + S (*p* < 0.05).

In the hippocampus of Study Group 2, Levene’s test confirmed homogeneity of variances (*p* > 0.05), and the effect size was η^2^ = 0.97. Hippocampal ATF4 levels were reduced in all groups vs. Naive (*p* < 0.001). R-SD + MEL had lower levels of ATF4 than R-SD + S (*p* < 0.001), R-SD + PBA (*p* < 0.01) and R-EC + MEL (*p* < 0.01). In the cortex, Levene’s test confirmed homogeneity of variances (*p* > 0.05), and the effect size was η^2^ = 0.94. Cortical ATF4 levels were higher in several groups (R-EC + S, R-EC + PBA, R-SD + S, R-SD + MEL) vs. Naive (*p* < 0.001), but they were lower in R-EC + PBA (*p* < 0.05) and R-EC + MEL (*p* < 0.001) vs. R-EC + S. R-EC + MEL had also lower levels of ATF4 than R-EC + PBA (*p* < 0.001). R-SD + PBA had significantly lower levels of ATF4 than all SD groups and R-EC + PBA (*p* < 0.001), and R-SD + MEL had significantly lower levels of ATF4 than R-SD + S (*p* < 0.05) and R-EC + MEL (*p* < 0.001).

#### ATF6

In the hippocampus of Study Group 1, Levene’s test confirmed homogeneity of variances (*p* > 0.05), and the effect size was η^2^ = 0.97. Hippocampal ATF6 levels were significantly lower in all groups compared to the Naive group (*p* < 0.001). EC + PBA and EC + MEL had significantly lower levels of ATF6 than EC + S (*p* < 0.01), and EC + MEL had significantly lower levels of ATF6 than EC + PBA (*p* < 0.001). SD + S had significantly lower levels of ATF6 than EC + S (*p* < 0.001). SD + PBA had higher levels of ATF6 than SD + S and SD + MEL (*p* < 0.05), but lower than EC + PBA (*p* < 0.001). SD + MEL had higher levels of ATF6 compared to EC + MEL (*p* < 0.05). In the cortex, Levene’s test confirmed homogeneity of variances (*p* > 0.05), and the effect size was η^2^ = 0.91. ATF6 levels were lower than Naive in all groups except SD + S (*p* < 0.001). EC + MEL had lower levels than EC + S and EC + PBA (*p* < 0.01). SD + MEL and SD + PBA had lower levels than SD + S (*p* < 0.001 and *p* < 0.01, respectively). SD + S had higher ATF6 levels than EC + S (*p* < 0.001), and SD + MEL had higher than EC + MEL (*p* < 0.01).

In the hippocampus of Study Group 2, Levene’s test confirmed homogeneity of variances (*p* > 0.05), and the effect size was η^2^ = 0.98. Hippocampal ATF6 levels were significantly lower in all groups compared to the Naive group (*p* < 0.001). R-EC + MEL had lower levels than R-EC + S and R-EC + PBA (*p* < 0.001). R-SD + MEL and R-SD + PBA had lower levels than R-SD + S (*p* < 0.001), and R-SD + MEL had also lower levels than R-SD + PBA (*p* < 0.01). Both R-SD + MEL (*p* < 0.01) and R-SD + PBA (*p* < 0.001) had lower ATF6 levels than the EC groups receiving the same treatment. In the cortex, Levene’s test confirmed homogeneity of variances (*p* > 0.05), and the effect size was η^2^ = 0.94. R-EC + PBA and R-EC + MEL had lower ATF6 levels than both the Naive and R-EC + S groups (*p* < 0.001). R-SD + PBA and R-SD + MEL had higher ATF6 levels than their corresponding EC groups (*p* < 0.001).

## Discussion

Sleep deprivation has been increasingly recognized as a significant contributor to cognitive impairment and neurodegenerative pathology. Our results indicate that REMSD may contribute to ER stress, AD-like pathological alterations, and deficits in spatial learning and memory. Notably, treatment with melatonin and the chemical chaperone 4-PBA mitigated these effects, suggesting their potential therapeutic value in sleep-related cognitive decline.

The cognitive deficits observed in REMSD animals align with previous studies showing that sleep loss disrupts hippocampal synaptic plasticity and long-term potentiation, essential processes for memory consolidation [34, 35]. While REMSD animals showed significant learning impairments during the learning phase, melatonin-treated animals demonstrated improved performance. This finding supports previous work showing melatonin’s ability to preserve hippocampal function under conditions of oxidative stress [36]. Interestingly, the probe test results showed that while recovery sleep alone could restore memory performance to control levels, animals receiving either melatonin or 4-PBA treatment exhibited superior retention, suggesting these compounds may enhance the restorative processes that occur during sleep. Similarly, 4-PBA’s ability to improve cognitive performance in neurodegenerative models [37] was corroborated in our study, reinforcing its role as an ER stress modulator.

The ER is essential for protein folding and trafficking. Conditions such as increased protein synthesis, energy deprivation, or inflammation can lead to the accumulation of unfolded proteins, causing ER stress. To counteract this, cells initiate the UPR, which has three stages: healthy, protective, and adaptive. At the molecular level, our study provides compelling evidence that REMSD triggers a robust ER stress response through multiple pathways. The significant increases in phosphorylated PERK observed in sleep-deprived animals indicate activation of the UPR. These findings corroborate previous reports demonstrating that sleep deprivation leads to accumulation of misfolded proteins and ER stress [24]. The temporal pattern of these changes is particularly noteworthy while acute sleep deprivation (6–12 h) induced marked increases in BiP levels, chronic sleep deprivation (7 days) resulted in more modest elevations, suggesting possible adaptive mechanisms or the onset of pathological changes that bypass normal stress responses [2, 38].

Melatonin is essential for circadian rhythm regulation and exhibits antioxidant, anti-inflammatory, and neuroprotective properties [39, 40]. It promotes neurogenesis and inhibits apoptosis [41]. Melatonin regulates redox homeostasis and reduces apoptosis following REMSD [32]. It decreases neuronal apoptosis by reducing BiP and CHOP expression in kainic acid-induced models [42] and promotes neuronal survival in intracerebral hemorrhage models by lowering ATF6 and CHOP levels while increasing Bcl-2 expression [43]. Chemical chaperones like 4-PBA alleviate ER stress and are used therapeutically [27, 44].

In Study Group 1, SD caused misfolded protein accumulation in the ER, reducing BiP levels. pPERK levels rose significantly, indicating ER stress activation. Early ER stress led to peIF2α, suppressing protein synthesis to prevent apoptosis. Prolonged stress, however, triggered eIF2α dephosphorylation, thereby increasing ATF4 translation and apoptosis-related proteins like CHOP.

In the cortex, pIRE1α levels increased due to REMSD, but melatonin and 4-PBA treatments reduced this effect, highlighting their role in ER stress modulation. Unlike the hippocampus, cortical ATF6 levels increased due to SD. Elevated IRE1 suggested an adaptive ER stress response. Melatonin increased peIF2α while reducing pIRE1, whereas 4-PBA only decreased pIRE1, indicating differential pathway effects.

Hippocampal samples showed elevated GRP94, a key ER chaperone, across all groups, confirming UPR activation. This response aids protein folding and reduces translation. Similarly, cortical GRP94 increased during SD but decreased with 4-PBA and melatonin, aligning with prior studies on ER stress during SD.

In Study Group 2 (recovery sleep analysis), hippocampal CHOP levels were significantly higher. ATF-6 and pIRE1 remained unchanged. Both melatonin and 4-PBA reduced CHOP, with melatonin also lowering pPERK, demonstrating stronger UPR modulation. Cortical ATF-4 levels were low in the SD group, with no other major differences, indicating recovery sleep’s restorative role. 4-PBA increased peIF2α and reduced CHOP, while melatonin decreased pPERK and CHOP while elevating peIF2α, underscoring its therapeutic potential.

Hippocampal GRP94 increased only in 4-PBA and melatonin-treated groups, implicating ER chaperones in UPR regulation during recovery sleep. Cortical results, however, showed no significant changes, suggesting that recovery sleep alone mitigated ER stress without additional treatment benefits.

Overall, treatments effectively countered ER stress during pathology, while recovery sleep independently alleviated cortical stress. Regional protein expression differences imply sleep’s restorative effects vary across brain areas. Melatonin and 4-PBA showed distinct regulatory impacts, with melatonin exhibiting broader UPR modulation. These findings highlight the complex interplay between ER stress pathways, therapeutic interventions, and sleep recovery mechanisms.

The differential effects of melatonin and 4-PBA on various ER stress pathways provide important insights into their mechanisms of action. Melatonin showed particularly strong suppression of the PERK-eIF2α-ATF4-CHOP pathway, which is associated with apoptosis under conditions of prolonged ER stress. This effect likely contributes to melatonin’s well-documented anti-apoptotic properties [43]. 4-PBA demonstrated more pronounced effects on the IRE1α-XBP1 pathway, consistent with its known function as a chemical chaperone that facilitates protein folding and reduces ER load [27].

Our findings regarding AD-related pathology are particularly significant in light of growing clinical evidence linking sleep disturbances to AD progression. The observed increases in hippocampal Aβ1–40 and Aβ1–42 following REMSD support the proposed role of sleep in amyloid clearance [10]. The glymphatic system, which is most active during sleep, has been shown to facilitate the removal of amyloid proteins from the brain [12, 13]. Our results demonstrate that both melatonin and 4-PBA can reduce Aβ accumulation, with melatonin showing particularly strong effects on Aβ1–42, the more aggregation-prone and neurotoxic species. This finding is consistent with previous studies showing melatonin’s ability to inhibit amyloidogenesis [45]. REMSD animals showed increased tau levels and GSK-3β activity, both of which were ameliorated by melatonin treatment. This supports the growing recognition that sleep disturbances contribute to tau hyperphosphorylation and tangle formation [11]. The mechanism likely involves multiple pathways, including melatonin’s inhibition of GSK-3β [46] and its modulation of protein phosphatase 2 A activity. The persistence of elevated tau in cortical tissues even after recovery sleep suggests that some tau-related changes may be less reversible than amyloid accumulation, potentially explaining why chronic sleep problems are associated with increased AD risk.

The regional differences observed in our study highlight that although both the hippocampus and cortex exhibited ER stress and AD-like alterations, the patterns of change and responses to treatment varied between these regions. The hippocampus, with its high metabolic demands and crucial role in memory formation, may be particularly sensitive to sleep-related disruptions in proteostasis. In contrast, cortical regions showed more persistent changes in some markers (e.g., Aβ1–40) even after recovery sleep. These regional variations may reflect differences in neuronal subpopulations, connectivity patterns, or susceptibility to specific types of stress.

Our apoptosis-related findings provide further evidence for the neuroprotective effects of melatonin. The increased Bax/Bcl-2 ratio and caspase-12 activation observed in REMSD animals are consistent with ER stress-induced apoptosis pathways [47]. Melatonin’s ability to modulate these markers supports its established role in regulating cell survival pathways [48]. The complex, region-specific effects on caspase activation (e.g., increased hippocampal caspase-12 in melatonin-treated SD animals versus decreased cortical levels) highlight the nuanced nature of melatonin’s actions, which may vary depending on cellular context and stress conditions.

Recent clinical investigations have provided translational support for the neuroprotective mechanisms of 4-phenylbutyrate (4-PBA) observed in preclinical models. It has been tested in several early-phase clinical trials for neurodegenerative diseases. In amyotrophic lateral sclerosis (ALS), the combination of 4-PBA and taurursodiol significantly slowed functional decline and prolonged survival in patients [49]. Additionally, dose-finding and tolerability studies in Huntington’s disease [50] as well as ongoing Phase I/II trials in Alzheimer’s and Parkinson’s diseases (e.g., NCT03533257, NCT02046434), have also been conducted. These emerging clinical data underscore the translational relevance of our findings and suggest that pharmacological chaperones such as 4-PBA hold therapeutic promise for mitigating neurodegenerative processes linked to ER stress and protein misfolding. While 4-PBA facilitates protein folding and alleviates ER stress via chaperone-like activity, melatonin’s protective effects operate through multiple pathways including MT1/MT2 receptor signalling, antioxidant/anti-inflammatory actions and potential modulation of ER-stress markers [51, 52]. Given these mechanistic differences, the two compounds are not directly interchangeable, and their distinct modes of action should be taken into account when interpreting protective effects in the REM sleep-deprivation model used here.

The present study has several limitations that should be acknowledged. First, only male rats were included to avoid variability associated with hormonal fluctuations across the estrous cycle; however, this prevents interpretation of sex-specific differences. Second limitation of this study includes possible off-target actions of 4-PBA, notably its inhibition of histone deacetylases (HDACs), which may contribute to changes in gene expression independent of ER stress For example, 4-PBA increases GLUT4 expression through HDAC5 inhibition in muscle cells [53], regulates cardiac differentiation via HDAC inhibition in embryonic stem cells [54] and promotes IL-8 expression by enhancing histone acetylation in gastric cancer cell lines [55]. Another limitation is the absence of electrophysiological validation (e.g., LTP or LTD recordings), which would clarify whether synaptic plasticity changes underlie the behavioral and molecular findings. A further limitation is that melatonin’s pleiotropic and dose-dependent effects were not dissected in this study; thus the relative contributions of its receptor-mediated vs. antioxidant/ER-stress-modulating actions remain undetermined. Finally, a direct mechanistic comparison between 4-PBA and melatonin, such as receptor antagonism studies, dose–response evaluation was not conducted, limiting the interpretation of how these agents differentially modulate neuroprotective pathways. Future studies integrating both sexes, electrophysiological assessments, and pathway-specific interventions will be essential to address current limitations and to delineate whether the effects of therapeutic agents converge or diverge at the molecular and functional levels.

In conclusion, REMSD impairs learning and memory, but post-deprivation treatment with melatonin and 4-PBA significantly mitigates these effects. Recovery sleep alone improved memory to control levels, but treated animals showed superior memory performance. These findings align with studies demonstrating the positive impact of melatonin and 4-PBA on learning and memory [36, 37]. Melatonin and 4-PBA have differential effects on various ER stress pathways, with melatonin showing superior efficacy in modulating the UPR. These findings highlight the potential therapeutic benefits of melatonin and 4-PBA in mitigating the cognitive and neurodegenerative effects of sleep deprivation.

## Data Availability

The data that support the findings of this study are available from the corresponding author upon reasonable request.
